# Is pain control for chronic neuropathic pain after inguinal hernia repair using endoscopic retroperitoneal neurectomy effective? A meta-analysis of 142 patients from 1995 to 2022

**DOI:** 10.1007/s00423-022-02748-6

**Published:** 2023-01-18

**Authors:** Stephanie Taha-Mehlitz, Anas Taha, Alex Janzen, Baraa Saad, Dana Hendie, Vincent Ochs, Lukas Krähenbühl

**Affiliations:** 1 Clarunis, University Centre for Gastrointestinal and Liver Diseases, St. Clara Hospital and University Hospital, 4002 Basel, Switzerland; 2 Department of Biomedical Engineering, Faculty of Medicine, University of Basel, 4123 Allschwil, Switzerland; 3 Department of Anesthesia, Greifswald University, 17489 Greifswald, Germany; 4 Faculty of Medicine, St George’s University of London, 2062 Nicosia, Cyprus; 5 Department of Surgery, College of Medicine, Sulaiman Al Rajhi University, Albukairyah, Saudi Arabia; 6 Roche Innovation Centre Basel, Department of Pharma Research & Early Development, 4070 Basel, Switzerland; 7 Oncosurgery Zurich, 8802 Kilchberg/Zurich, Switzerland

**Keywords:** Endoscopic approach, Retroperitoneal neurectomy, Chronic neuropathic pain, Minimally invasive surgery, Hernia repair

## Abstract

**Purpose:**

Neuropathic pain is a complication after groin hernia surgery. Triple neurectomy of the iliohypogastric nerve, ilioinguinal nerve and genitofemoral nerve is an efficient treatment modality, with several surgical approaches. The minimally invasive endoscopic method to neurectomy was specifically investigated in this meta-analysis. Our aim is to determine the efficacy of this method in the treatment of chronic neuropathic pain posthernia repair surgery.

**Methods:**

A systematic review was conducted using four databases to search for the keywords (“endoscopic retroperitoneal neurectomy” and “laparoscopic retroperitoneal neurectomy”). The NCBI National Library of Medicine, Cochrane Library, MEDLINE Complete and BioMed Central were last searched on 26 May 2022. Randomised control trials and retrospective or prospective papers involving endoscopic retroperitoneal neurectomy operations after inguinal hernia repair were included. All other surgeries, procedures and study designs were excluded. The internal quality of included studies was assessed using the Newcastle–Ottawa Scale. The percentage of patients who had reduction in pain (“positive treatment outcome”) was used to assess the procedure’s effectiveness in each analysis.

**Results:**

Five comparable endoscopic retroperitoneal neurectomy studies with a total of 142 patients were analysed. Both the Wald test (*Q* (6) = 1.79, = .775) and the probability ratio test (*Q* (6) = 4.24, = .374) provide similar findings (0.000, 0.0% [0.0%; 78%]). The meta-analysis’ key finding is that the intervention was up to 78% effective (95% confidence interval, 71%; 84%).

**Conclusion:**

Endoscopic retroperitoneal neurectomy can be an effective treatment option for postoperative neuropathic pain relief following surgical hernia repair. Although there is limited reported experience with this technique, it may provide a clinical benefit to the patient. We recommend further prospective data and long-term follow-up studies be conducted to confirm and expand on these outcomes.

## Introduction

Chronic pain, defined as pain that lasts for three to 6 months and frequently fluctuates, is a recognized complication in patients after groin hernia repair [1]. In the literature, it has been documented that 0.5–37% of patients experience chronic pain after surgical groin hernia repair [2–7].

Chronic neuropathic pain, also known as persistent pain with characteristics like burning or shooting, can occur due to damage to the somatosensory system [1]. Modern open and laparoscopic repairs of groin hernias can cause entrapment-related symptoms from the genital nerves, specifically the genitofemoral and ilioinguinal [4]. Usually, such symptoms are self-limiting within a few weeks following the operation and so require no extended therapy [4]. However, persistent severe symptoms could lead to substantial morbidity and surgical reintervention. It is reported these symptoms are less likely to occur and less likely to be severe using minimally invasive methods [8].

Chronic neuropathic pain significantly influences activity and comfort levels, negatively impacting the patient’s quality of life. It has also been linked to psychiatric illnesses, including anxiety and depression, which may impact a patient’s well-being [9, 10]. Conservative management of neuropathic pain can include pharmacological, interventional and behavioural therapy while surgical options were devised to alleviate recurrent and refractory pain [9, 10]. Triple neurectomy of the ilioinguinal nerve (IIN), iliohypogastric nerve (IHN) and genitofemoral nerve (GFN) has emerged as a viable therapy option for this debilitating pain [3].

Despite the fact that case reports and other retrospective studies exist for open, laparoscopic, retroperitoneal and transperitoneal endoscopic techniques to triple neurectomy [11, 12], this meta-analysis focuses on the least invasive retroperitoneal endoscopic method. Our aim is to determine the efficacy of this method in the treatment of chronic neuropathic pain posthernia repair surgery. To the best of our knowledge, this is the first meta-analysis for this specific minimally invasive surgery.

### Anatomy

**Fig. 1:**
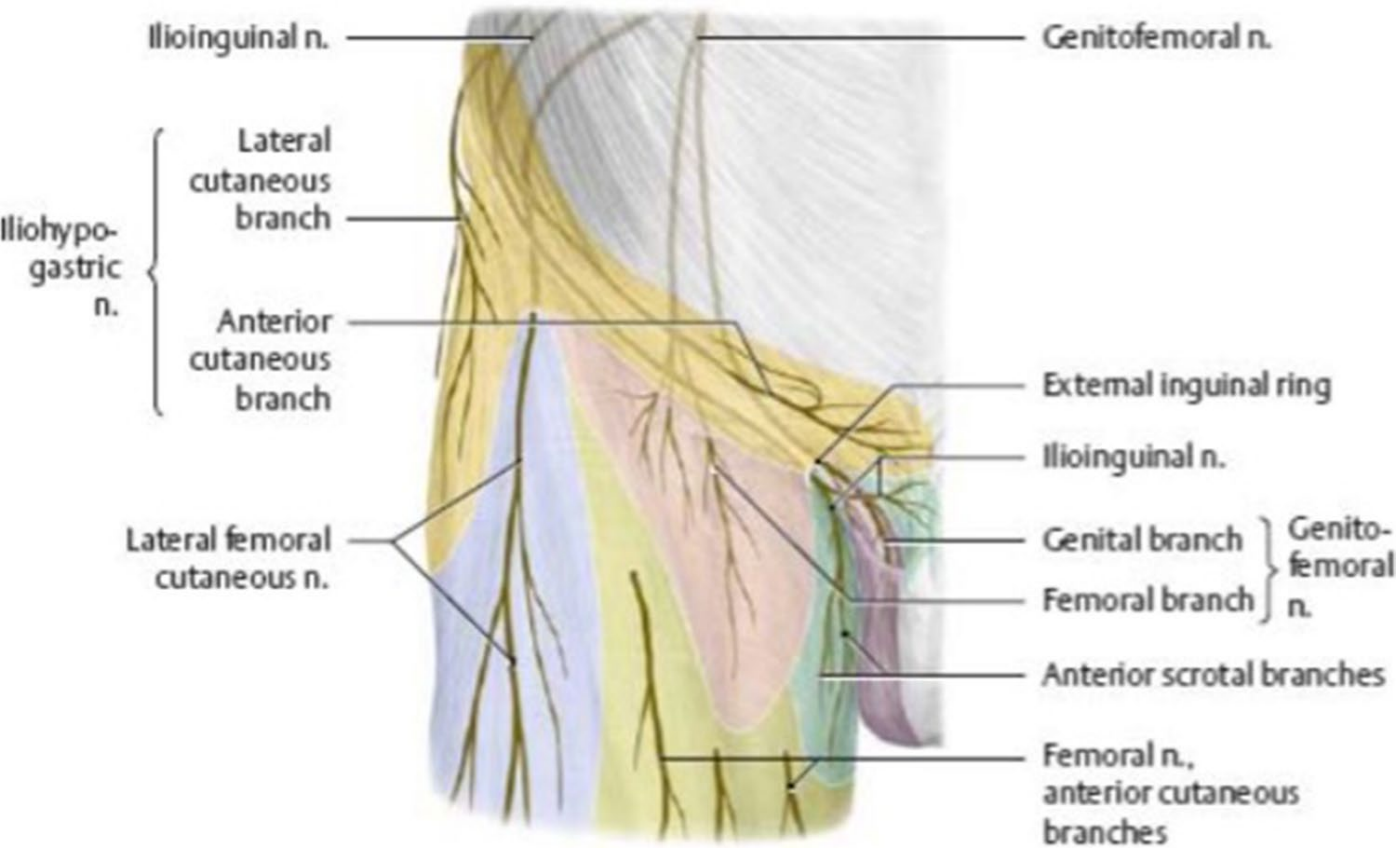
Sensory innervation of the inguinal region, according to https://doctorlib.info/medical/anatomy/29.html

Endoscopic retroperitoneal neurectomy necessitates a thorough understanding of the IHN, IIN and GFN locations and distributions. The lumbar plexus, represented by T12–L5, gives origin to these three nerves. The IHN, IIN and GFN are attributed to the lumbar plexus and contain parts of T12–L2. All three nerves (Fig. 1) include motor and sensory fibres and pass via the retroperitoneal space. The retroperitoneal space is bounded ventrally by the parietal peritoneum, dorsally by the fascia of the greater psoas muscle, laterally by the transverse fascia and cranially by the diaphragm. Caudally, it continues as the connective tissue of the lesser pelvis [5, 13].

The origins of the IHN are the segments T12–L1. These cords unite to form the IHN and run dorsally to the psoas major muscle on the quadratus lumborum muscle. After penetrating the transversus abdominis muscle, it follows the inguinal ligament medially. Above the superficial inguinal ring, it goes through the external oblique muscle. Its motor fibres innervate the caudal and lateral abdominal muscle, while its cutaneous rami (a branch of a nerve or blood vessel) innervate the skin above the inguinal ligament and the lateral hip region [11, 12, 14, 15].

The IIN is formed by rami anteriores from T12 to L1. The nerve course is analogous to the IHN from the iliac crest to the inguinal ligament medially but breaks through the superficial inguinal ring directly entering the inguinal canal. The motor fibres of the nerve innervate the same areas as those of the IHN. Moreover, it innervates the skin of the penile root or labia majora, with its scrotal or labial anterior nerves [11, 12, 14, 15].

The GNF consists of rami anteriores from the segments L1 and L2. After penetrating the psoas major muscle, it divides into femoral and genital rami. The former travels to the saphenous hiatus by passing through the lacuna vasorum. In men, the genital ramus is part of the spermatic cord in the inguinal canal and innervates the motor portion of the cremasteric reflex and the skin of the scrotum. In women, it supplies the labia majora as it passes through the round ligament. The femoral ramus of the GFN innervates the skin of the medial side of the thigh regardless of gender [11, 12, 14, 15].

### Surgical technique of endoscopic retroperitoneal neurectomy

There are many surgical methods allowing for a successful neurectomy of the IHN, IIN and GFN. While analyses of open, laparoscopic transperitoneal and endoscopic procedures for triple neurectomy have been published [16, 17], this meta-analysis focuses on the endoscopic retroperitoneal approach, described in detail in the *British Journal of Surgery* in 1997 [5].

**Fig. 2:**
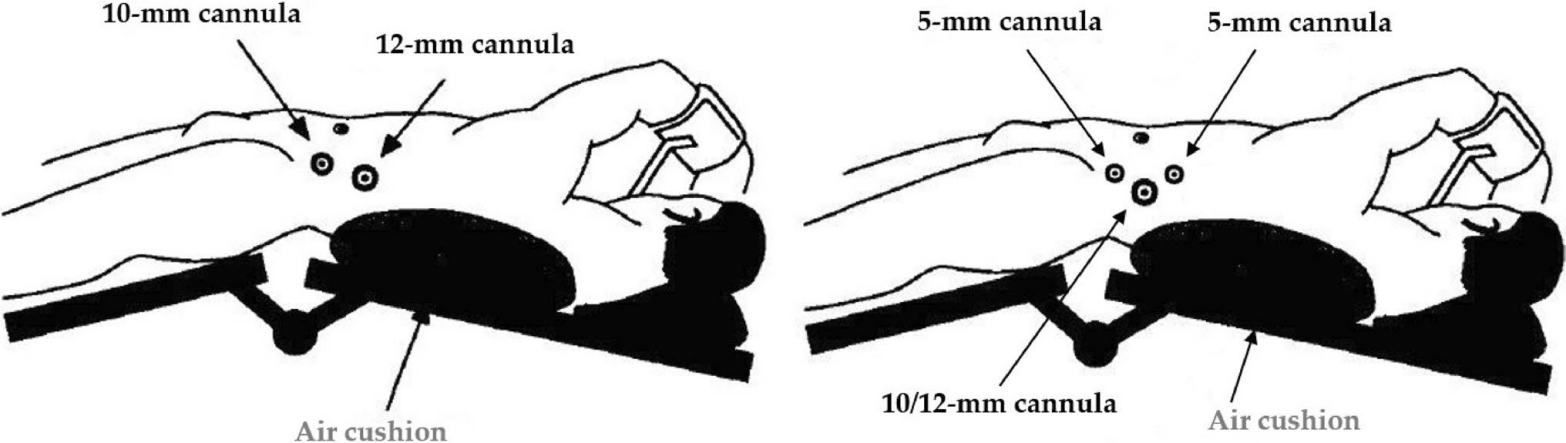
Surgical positioning of the patient and access route for endoscopic visualization of the retroperitoneal space. As described in [4, 5, 18, 34]; modified with permission [18]. Left: initial technique described by Krähenbühl et al. [5] for the first time in 1997. Right: modified technique with two 5 mm cannulae and one 10 mm or 12 mm cannula

The process begins with an intravenous antibiotic prophylaxis administered before the surgery. The patient undergoes general anaesthesia and is placed in a supine, slightly lateral posture (Fig. 2). Then, a transverse incision is made midway between the costal border and the anterior superior iliac spine in the midaxillary line. A 12 mm cannula is placed through this incision, and carbon dioxide is insufflated at a pressure of 12 mmHg. Under direct vision, cannulae of 10 mm and 5 mm are placed. Furthermore, a 4–5 cm dissection of the GNF, including the bifurcation, is performed at the level of the psoas muscle. When the IHN/IIN are dissected, the nerves are exposed at the area where they cross the quadratus lumborum muscle and then excised proximally and laterally to the GFN. Furthermore, all resected nerve specimens are subjected to histological examination [18].

## Methods

### Search strategy

The authors A. J., A. T. and D. H. conducted independent searches for literature using the NCBI National Library of Medicine, Cochrane Library, MEDLINE Complete and BioMed Central. The date of the last search was 26 May 2022. The following single-text terms were used in the search strategy: “endoscopic retroperitoneal neurectomy” and “laparoscopic retroperitoneal neurectomy”. The retrieved studies’ reference lists and citations were manually searched for more relevant research.

### Inclusion and exclusion criteria

The search technique was based on an orderly structure of core phrases, and only papers involving endoscopic retroperitoneal neurectomy operations after inguinal hernia repair were included. Randomised control trials and retrospective or prospective study designs were included in this study. Exclusion criteria included case reports, case series, comment letters, studies with poor design such as studies with insufficient data and studies with Newcastle–Ottawa quality assessment score of 4 or less [19]. All other surgeries, including open and laparoscopic approaches to neurectomy, as well as any procedures that follow a transperitoneal approach, were excluded.

### Data collection

After the removal of duplicates, full-text evaluation and data extraction was completed by two independent authors. The internal quality of included studies was assessed using the Newcastle–Ottawa Scale by two independent authors [20].

### Data items and variables

The percentage of patients who had received significant pain reduction postoperatively, in addition to the frequencies and proportions of therapy (“proportion of positive results”), were the primary outcome values assessed in this meta-analysis. Pain reduction was defined as statistically significant pain reduction based on pre- and postoperative questionnaire scores. Questionnaires used in analysed studies include the following: NRS-11: Numeric Rating Scale; I-PROM: Individual Patient-Reported Outcome Measure; NRS: Numerical Rating Scale; PDQ: PainDETECT Questionnaire [3, 4, 8, 18, 21].

### Statistical analysis

Statistical analysis was conducted using the R programme Meta (G. Schwarzer, version 4.18–0 from March 5, 2021). The Wald test and the probability ratio test were used to analyse statistical heterogeneity. Furthermore, for the frequencies of positive treatment outcome occurrence, a logistic regression model with a random intercept and a maximum-probability estimator of 2 and the Hartung-Knapp adjustment for random effects models were employed to analyse the outcomes. The model’s outputs were used to calculate the logits of the frequencies’ chances. Due to the fact that the treatments in three separate studies were all positive, the inclusion of the three separate studies in the model was made possible using a continuity correction of 0.5. The findings were accompanied by their 95% Clopper-Pearson confidence intervals. Furthermore, a *p* < 0.05 was considered statistically significant. In addition, a funnel plot and a radial plot were developed to detect publication bias, and other statistical tests such as a rank correlation test and three regression tests were utilised.

## Results

### Excluded studies

**Fig. 3:**
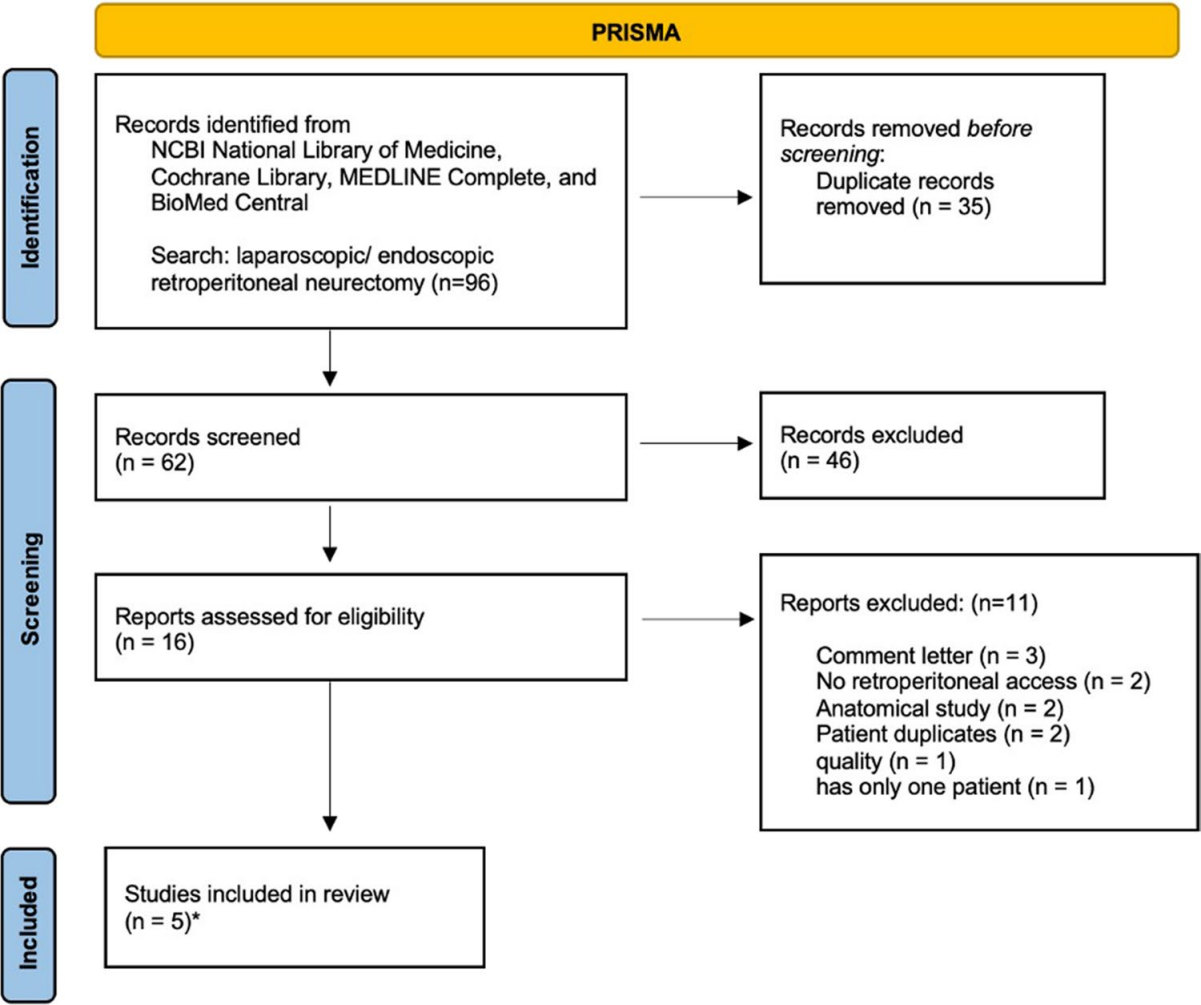
Flow diagram of the review process. *Not all patients in these studies were included in the analysis for the following reason (unrelated to chronic pain after hernia repair *n* = 8, open surgery *n* = 33)

The initial literature search was carried out, with a specific filter set to display full-text articles. As a result, 97 records were discovered, which were reduced to 62 when duplicates were deleted. As a result, 46 papers were omitted because they did not describe surgical procedures involving retroperitoneal surgical access and hence did not match the inclusion criteria. The full text of 16 articles was reviewed. As a consequence, 11 more papers (Table 1) were removed for the following reasons: one for a lack of quality according to the Newcastle–Ottawa Scale [19], three for being comment letters, two for employing an intraabdominal surgical technique, two for being anatomical investigations and one for having just one patient. Two studies were omitted because they contained the same patients as other included research; see Fig. 3.

**Table 1:** Excluded studies

Excluded studies	Reason
Song JW, Wolf JS Jr, McGillicuddy JE, Bhangoo S, Yang LJ. (2011)	Only one patient
George, Williams, Franklin, and Dellon (2019)	NRA
Narita M, Jikihara S, Hata H, Matsusue R, Yamaguchi T, Otani T, Ikai I. (2017)	Quality
Vuilleumier H, Hübner M, Demartines N (2010)	Comment letter
Troidl H (1997)	Comment letter
Schoeller T, Wechselberger G, Otto A, Lille S. (1997)	Comment letter
Moreno-Egea A. (2021)	Anatomical study
Karampinis I, Weiss J, Pilz L, Post S, Herrle F. (2017)	NRA
Geh N, Schultz M, Yang L, Zeller J. (2015)	Anatomical study
Chen, Hiatt, and Amid (2013)	Duplicated patient
Krähenbühl, Striffler, Baer, and Büchler (1997)	Duplicated patient

*NRA* no retroperitoneal access

### Study selection

**Fig. 4:**
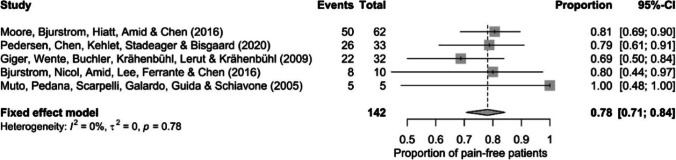
Forest plot presenting the frequencies and proportions of treatment and their 95% Clopper-Pearson confidence intervals

All publications about the minimally invasive endoscopic retroperitoneal approach to inguinal triple neurectomy were included. Five studies were found, with 142 patients of both genders who received successful surgery over a 24-year span. It should also be highlighted that the initial number of patients was 182, but 41 individuals from the five studies were excluded from the analysis, since 8 had persistent pain unrelated to prior hernia surgery and 33 had open neurectomies. These five papers were appropriate for meta-analysis and systematic review (Fig. 4) [3, 4, 8, 18, 21]. The study characteristics are described and summarized in Table 2.

**Table 2:** Study characteristics

Overall cohort							
Study	Location	Design	Study period	Size (*n*)		Mean age (years)	Male (%)
Moore et al. 2016	USA	Retrospective cohort	2012–2015		62	47	51 (82%)
Pedersen et al. 2020	Denmark	Prospective cohort	2016–2019		33	48	31 (94%)
Giger et al. 2009	Switzerland	Retrospective cohort	1997–2007		32	47	28 (88%)
Bjurstrom et al. 2016	USA	Prospective cohort	2014–2015		10	47	9 (90%)
Muto et al. 2005	Italy	Retrospective cohort	2002–2005		5	NA	5 (100%)

### Examination of quality bias

Of the 5 studies included, 100% scored 5 or higher on the Newcastle–Ottawa Scale indicating good quality studies (Table 3) [19].

**Table 3:** Newcastle–Ottawa Scale assessment of quality and bias

Newcastle–Ottawa Scale										
Study ID	Selection				Comparability	Outcome			Total score (max = 10)	Power
	Representativeness of the exposed cohort (max = *)	Selection of the non-exposed cohort (max = *)	Ascertainment of exposure (max = *)	Demonstration that outcome of interest was not present at start of study (max = *)	Comparability of cohorts on the basis of the design or analysis (max = **)	Assessment of outcome (max = *)	Was follow-up long enough for outcomes to occur (max = *)	Adequacy of follow-up of cohorts (max = *)		
Moore, 2016	*	*	*	-	-	*	*	*	**6**	**Good quality**
Pedersen, 2020	*	*	*	-	-	*	-	*	**5**	**Good quality**
Giger, 2009	*	*	*	-	-	*	-	*	**5**	**Good quality**
Bjurstorm, 2016	*	*	*	-	-	*	*	*	**6**	**Good quality**
Muto, 2005	*	*	*	-	-	*	*	*	**6**	**Good quality**

### Systematic review and meta-analysis

The distribution of the findings was highly homogenous (*τ*^2^ = 0.000, *I*^2^ = 0.0% [0.0%; 70.8%]). The Wald test (*Q* (6) = 1.79, *p* = 0.775) and the probability ratio test (*Q* (6) = 4.24, *p* = 0.374) did not reveal any significant differences. The meta-analysis was carried out using a fixed effects logistic regression model due to the homogeneity of the research data. Figure 4 shows the frequencies and proportions of treatment and their 95% Clopper-Pearson confidence intervals.

Successful treatment was defined as a significant reduction in pain. The meta-analysis shown concluded that the surgical treatment across all individual studies was 78% successful (95% confidence interval, 71%: 84%) with 111/142 patients experiencing reduced pain. According to the 95% confidence interval, the treatment was successful in at least 71%, meaning in at least two-thirds of cases.

### Examination of publication bias

**Fig. 5:**
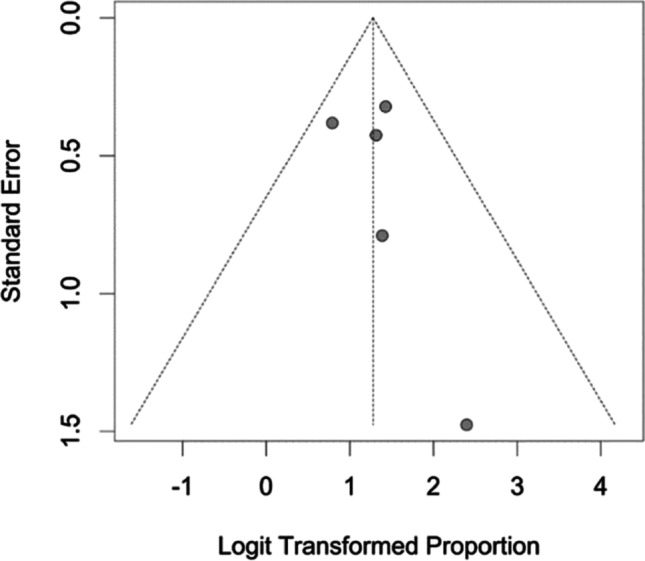
Funnel plot of the meta-analysis

Figure 5 shows a funnel plot which was generated to see if the results may be skewed by a publishing bias. An odds of 1 and hence a 50% success rate for therapy are indicated on the graph, with the x-axis showing logit-transformed proportions. The absence of small studies with non-positive and non-significant outcomes suggests a publishing bias in this compilation.

**Fig. 6:**
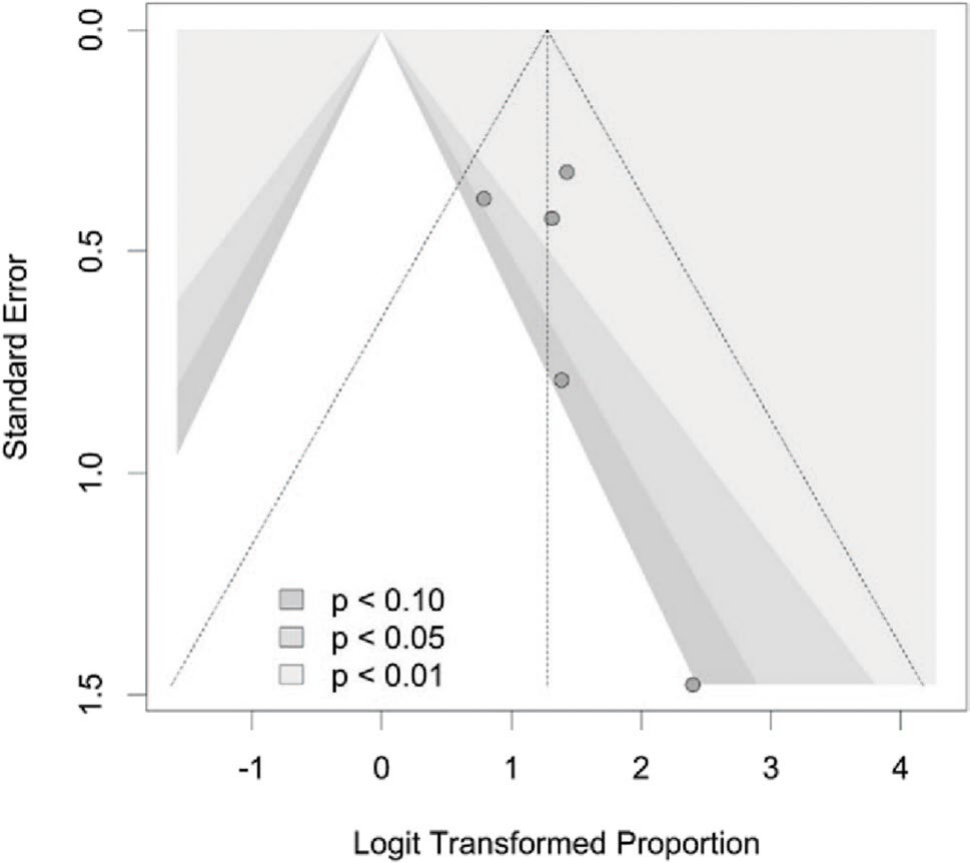
Funnel plot with significant range

In Fig. 6, contour lines for several significance levels have been inserted [22]. Non-significant findings do not appear to be concealed in our analysis as is typical in cases of publication bias. There are two small and two major studies with non-significant findings at the 5% level.

**Fig. 7:**
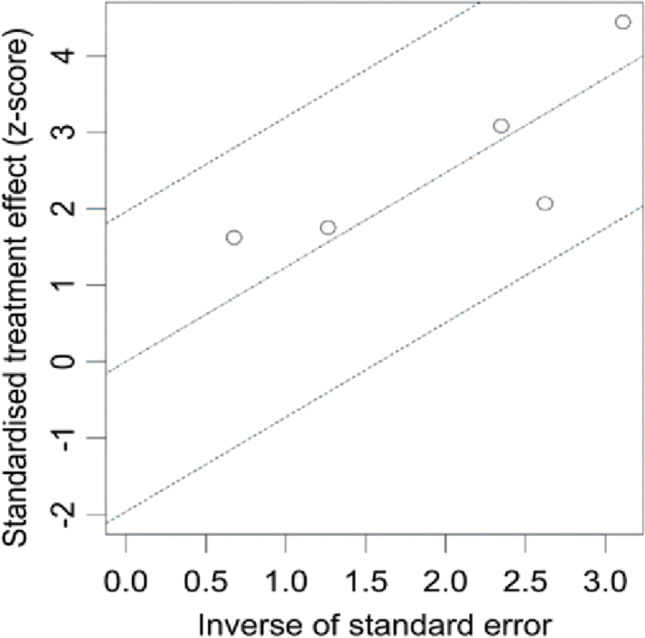
Radial plot of meta-analysis

Figure 7 depicts a radial map showing the findings of individual investigations [23]. All studies scatter more or less randomly in the 95% confidence interval and around the middle regression line if the treatment effect is 75%, so that the image does not show any red flags for publication bias.

### Radial plot for meta-analysis

In addition, four statistical tests were conducted to check the asymmetry of the funnel plot, the results of which are summarized in Table 4. These four tests are the rank correlation test by Begg and Mazumdar [24], the regression test by Egger et al. [25], the regression test by Thompson and Sharp [26] and the regression test by Peters et al. [27]. The tests all lead to the conclusion that the asymmetry of the funnel plot must be viewed as random and that there is no evidence of a publication bias.

**Table 4:** Results of the statistical tests on the asymmetry of the funnel plot

Scheme 18	Results
Rank correlation test of funnel plot asymmetry according to Begg and Mazumdar [24]	*z* = 0.49, *p* = .624
Linear regression test of funnel plot asymmetry according to Egger [25]	*t*(5) = 0.79, *p* = .486
Linear regression test of funnel plot asymmetry according to Thompson and Sharp [26]	*t*(5) = 0.65, *p* = .564
Linear regression test of funnel plot asymmetry according to Peters et al. [27]	*t*(5) = − 0.14, *p* = .898

## Discussion

This systematic review was conducted to characterise the success rate of endoscopic triple neurectomy and thus provide a valid recommendation whether this technique should be considered for patients with postoperative inguinal neuropathic pain. Furthermore, the literature on this technique can be considered valid as thorough statistical evaluation did not reveal any indicators that the collected data might have significant publication bias. Of the 142 patients from five studies who underwent endoscopic triple neurectomy for the indication mentioned above, approximately 75% reported complete or at least significant reduction in subjective pain intensity.

Even though its exact occurrence rate remains controversial, there is consistent evidence that postoperative neuropathic pain has surpassed hernia recurrence as the most frequent complication of hernia repair [28–30]. However, surgical denervation after hernia surgery is not indicated in all cases. While most patients with chronic pain can be managed conservatively with medication or therapy, it does not always prove to be straightforward. Patients may have to deal with central nervous system side effects or dependence and still not achieve sufficient pain relief [31].

Triple neurectomy of the IIN, IHN and GFN has been proven to be an excellent treatment method in cases of chronic pain. Stulz and Pfeiffer [32] first described the procedure in 1982 as an ultima ratio method for neuropathic pain caused by nerve entrapment following surgeries in the lower abdomen. Over the years, different surgical techniques for neurectomy were established including open, laparoscopic and endoscopic approaches [10, 18, 33]. Combined procedures, such as open with laparoscopic or open with endoscopic procedure, have been described in the literature [16, 17]. According to several authors, the success rate of these interventions is 70–100% [2]. Only orchialgia does not significantly improve after this intervention, which could be related to the more complex innervation of the gonads [34, 35].

Postoperatively, numbness might be felt over the treated area, a possibility that should be explored and discussed with the patient [34]. A small number of side effects have been described after undergoing these procedures. Among them are loss of cremaster reflex and hypoesthesia in the labia majora, scrotum and skin of the femoral region [34]. However, the significant pain reduction experienced by patients usually outweighs the possible adverse outcomes. The actual frequency of these complications seems to be less than expected. Among 142 patients reviewed in this study, none developed any of the aforementioned complications postoperatively [21]. Triple laparoscopic neurectomy consists of an “open two-stage operation”, which is also performed in patients with chronic groin pain. This method is equally minimally invasive but requires adequate knowledge of the anatomical variations of the genitofemoral nerve and the overall neuroanatomy of the genital and inguinal regions [9]. The success rate of triple laparoscopic neurectomy is much higher than that of the standard procedure with all three nerve branches transected [34].

Another method that may be used if traditional laparoscopic surgery is not feasible is the “dual two-team approach”. It combines open surgical and endoscopic techniques. Although this method showed promise with high success rates on a comparative scale, no definitive conclusion can be drawn due to only one small descriptive study discussing this method [16].

Endoscopic retroperitoneal neurectomy is a one-stage, minimally invasive procedure for resecting the IHN, IIN and GFN from the lumbar plexus. This method has been found to provide excellent pain relief and morbidity rates comparable to open neurectomy, but it is a challenging procedure even in the hands of experts, and anatomical variances make the identification of the nerves even more complex [18].

The retroperitoneal access may present some inconveniences inherent to any surgical procedure, including extensive dissection, painful incision and complications such as surgical wound infection, hematoma, bleeding, incisional hernia and postoperative paralytic ileus. In the population of 142 patients, 3 patients had complications due to the procedure. Two patients had a laceration of the posteroinferior diaphragm requiring laparoscopical suturing and one patient had a delayed haemorrhage requiring transfusion and embolization. No other complications were reported intra- or perioperatively in the population. The advantage of this technique is providing broad access to the retroperitoneal cavity and efficient control of haemorrhage. This procedure is described to be less painful and provides faster postoperative recovery, discharge and earlier return to usual activities [3]. As the subject of this meta-analysis, we evaluated exclusively endoscopic retroperitoneal neurectomy to treat patients with persistent neuropathic pain. Unlike open neurectomy, the endoscopic method proved to be the procedure with the least invasive approach and simultaneously similarly effective in pain relief [18]. While the endoscopic retroperitoneal technique allows visualization of all nerves of the lumbar plexus, the open surgical triple neurectomy provides only identification of the genitofemoral nerve with certainty [18].

There are several limitations to this study. First, this review included articles that contained various methods for quantifying chronic neuropathic pain in posthernia repair surgery. Second, the number of patients analysed is small due to the paucity of published literature on this specific approach which will increase the number of type II errors. Third, the total length of follow-up in the studies included was between 3 months and 1 year. Longer follow-up in future studies will help better understand long-term quality-of-life assessments postprocedure. Large, multicentered prospective and randomised control studies using standardized treatments and well-defined pain outcome measurements must be performed in order to increase the predictive power of postoperative outcomes. This surgical technique is the last line of treatment meaning there can be a learning curve and reduced feasibility in practical application [3]. Our analysis, along with future research, should drive a standardized protocol that can improve both the reproducibility and feasibility of such a method.

## Conclusion

Persistent neuropathic pain is a common complication of surgical hernia repair. However, it is often debilitating as it permanently affects the patient’s physical abilities and mental health. As a result, several techniques have been developed to relieve the symptoms of postoperative inguinal neuropathic pain. We conducted a meta-analysis of five studies on the minimally invasive endoscopic retroperitoneal method for ilioinguinal, iliohypogastric and genitofemoral nerve triple neurectomy. Our analysis showed that endoscopic retroperitoneal neurectomy can be an effective treatment option for postoperative neuropathic pain relief following surgical hernia repair. Considering the small number of patients in this study, data interpretation with regard to efficacy can only be limited. Although there is limited reported experience with this technique, it may provide a clinical benefit to the patient. Further prospective data and long-term follow-up of the triple neurectomy procedure will be needed to confirm these outcomes.
